# A Review of Therapeutic Agents Given by Convection-Enhanced Delivery for Adult Glioblastoma

**DOI:** 10.3390/ph17080973

**Published:** 2024-07-23

**Authors:** Nathaniel W. Rolfe, Nicholas B. Dadario, Peter Canoll, Jeffrey N. Bruce

**Affiliations:** 1 Department of Neurological Surgery, Columbia University Irving Medical Center/NY-Presbyterian Hospital, New York, NY 10032, USA; nbd2122@cumc.columbia.edu; 2 Department of Pathology and Cell Biology, Columbia University Irving Medical Center/NY-Presbyterian Hospital, New York, NY 10032, USA; pc561@cumc.columbia.edu

**Keywords:** convection-enhanced delivery, drug delivery, glioblastoma, high-grade glioma, blood-brain barrier, volume of distribution

## Abstract

Glioblastoma remains a devastating disease with a bleak prognosis despite continued research and numerous clinical trials. Convection-enhanced delivery offers researchers and clinicians a platform to bypass the blood–brain barrier and administer drugs directly to the brain parenchyma. While not without significant technological challenges, convection-enhanced delivery theoretically allows for a wide range of therapeutic agents to be delivered to the tumoral space while preventing systemic toxicities. This article provides a comprehensive review of the antitumor agents studied in clinical trials of convection-enhanced delivery to treat adult high-grade gliomas. Agents are grouped by classes, and preclinical evidence for these agents is summarized, as is a brief description of their mechanism of action. The strengths and weaknesses of each clinical trial are also outlined. By doing so, the difficulty of untangling the efficacy of a drug from the technological challenges of convection-enhanced delivery is highlighted. Finally, this article provides a focused review of some therapeutics that might stand to benefit from future clinical trials for glioblastoma using convection-enhanced delivery.

## 1. Introduction 

Despite more than 1300 clinical trials for glioblastoma (GBM) over the last twenty years, there has been only marginal progress in combatting GBM’s bleak prognosis [1]. Numerous phase 1 and 2 trials of promising preclinical drugs have failed to show significant improvement in clinical outcomes, and high-grade gliomas remain nearly universally fatal. This failure is made all the starker given the steady progress made in long-term survival for a variety of non-CNS cancers [2]. The failure of promising preclinical drugs is due in large part to GBM’s inter- and intra-tumoral heterogeneity, dose-limiting side effects, and inadequate penetration of the blood–brain barrier (BBB), particularly along the non-enhancing, infiltrative margin of the tumor [3].

A major challenge in developing a novel GBM therapy is reaching a therapeutic concentration at the tumor margins. While small, lipophilic molecules like carmustine and temozolomide reach therapeutically effective concentrations within the brain parenchyma, the BBB excludes almost all high molecular weight drugs and the vast majority of low molecular weight compounds [4]. It is estimated that less than 5% of existing drugs can pass the BBB to a clinically relevant degree [5]. The challenges related to the delivery of drugs across the BBB are well known and have spurred various physical (e.g., convection-enhanced delivery and focused ultrasound) and chemical (e.g., ABC transport inhibitors, intelligent prodrug design) approaches to bypass the BBB [6].

Some recent studies have shown that the tumor environment alters BBB integrity, leading to what is often referred to as the blood–tumor barrier (BTB) [7]. While the implication of a leaky BTB is that it may allow the passage of previously non-penetrating drugs, the heterogenous nature of BBB breakdown in brain tumors appears to prevent the accumulation of optimal and consistent drug concentrations throughout the tumor [6,8]. 

Even when a drug crosses the BBB, efflux pumps, intraparenchymal metabolism, and leakage into lymphatics and CSF all limit concentrations in the brain parenchyma compared to peripheral organs [9]. To counter this, an effective oral or intravenous treatment for GBM often requires increased dosing, leading to high rates of systemic toxicity [10]. Temozolomide (TMZ) illustrates this concept; concentrations in CSF are only 20% of plasma concentrations, and higher or prolonged dosing is prevented by elevated rates of high-grade myelosuppression [11,12]. Systemic toxicities have also stymied clinical trials of new GBM treatments [13,14,15,16].

Given the unique challenges inherent to delivering therapeutics to brain cancer cells via the blood, several strategies have evolved in the translational setting to help deliver high concentrations of various drugs into the brain parenchyma. An overview of such techniques can be found elsewhere, but one promising technique to circumvent both the BBB and potential systemic toxicities is convection-enhanced delivery (CED) [6,9].

Since its development in the 1990s, CED has offered researchers and clinicians a platform to bypass the BBB and deliver therapeutics directly into the brain parenchyma [17,18]. CED utilizes stereotactically placed catheters to deliver a continuous positive-pressure micro-infusion of drugs into the brain. As such, CED bypasses the BBB and allows for targeted delivery of infusate to the peritumoral area, an attractive feature given the tendency of GBM to recur near the tumor margins [19]. Local delivery of anticancer agents also limits systemic side effects as drug concentrations are negligible outside the brain parenchyma. With CED, dose-limiting toxicities are neurologic, as opposed to nephrotic and hematologic, as are most often seen with systemically delivered chemotherapy [20].

In CED, the tissue distribution of a therapeutic agent is primarily a function of the pressure gradient as opposed to the concentration gradient [21]. The consequence is that CED allows for homogenous concentrations over a wider volume of distribution than can be achieved via passive diffusion. In theory, CED allows a wide range of water-soluble compounds to be delivered to the CNS. Not only can small-molecule therapeutics be delivered, but proteins, viruses, nanoparticles, oligonucleotides, and antibodies can all be infused into the extracellular space of the CNS [22]. Distribution of therapeutics by CED is not unlimited, however, as particle size, charge, binding capacity, and heterogeneity of the porous, extracellular brain all influence CED drug distribution [23,24]. Also, drugs that easily pass the BBB are poor candidates for CED as they are able to quickly exit the interstitium and cross into systemic circulation. Similarly, drugs that are rapidly metabolized in the CNS or sequestered by healthy nervous tissue would have limited benefit when given by CED.

Given the wide range of therapeutic compounds that can be delivered via CED, we believe that an updated review of agents used in past adult GBM clinical CED trials would be beneficial. In addition, we will provide a focused review of some of the therapeutics that might stand to benefit from delivery by CED with an eye toward what the future of CED might look like. The clinical trials of CED reviewed in this article are shown in Table 1, which represents a comprehensive summary of CED clinical trials for adult GBM patients to date. 

## 2. Antitumor Agents Delivered by CED in GBM Clinical Trials

### 2.1. Conjugate Toxins

Early clinical trials of CED, beginning with Laske et al. in 1997, used conjugate protein toxins as their therapeutic agent [25,29,33,34]. This early focus on protein-toxin conjugates is likely a consequence of increased interest in the 1980s and 1990s regarding the creation of “biological missiles”, with antibodies or receptor ligands targeting potent toxins to cancerous cells [87]. Early research investigated the potential of diphtheria toxin and pseudomonas exotoxin, two bacterially secreted toxins that potently inhibit elongation factor 2 (EF2) [88,89]. 

#### 2.1.1. Tansferrin-CRM107 (Tf-CRM107)

Transferrin-CRM107 combines human transferrin (Tf) to a diphtheria toxin via a stable thioether bond [26]. The diphtheria toxin moiety contains two point mutations that decrease non-specific human cell binding but leave intact the catalyst function of the protein (subunit A). Subunit A inhibits protein synthesis by catalyzing the attachment of adenosine diphosphate ribose to EF2 [26]. Human transferrin is attached because transferrin receptors are overexpressed on rapidly dividing cells, including in GBM [27]. In vitro studies showed that the attachment of the transferrin moiety led to an approximately 5-fold improvement in IC_50_ values [33].

In vivo mouse studies showed that Tf-CRM107 inhibited U251 glioma flank growth in nude mice in a dose-dependent manner [28]. Based on this promising result, phase I and phase II trials of Tf-CRM107, delivered by CED, were conducted (Laske et al. and Weaver et al.) [25,33]. The safety of Tf-CRM107 was shown alongside a response rate of 35% in recurrent GBM and anaplastic astrocytoma patients, which correlated with favorable survival [33]. The most common serious adverse event was symptomatic cerebral edema (14% of patients), which correlated with MRI changes suggestive of venous thrombosis. This was hypothesized to be partially due to higher expression of the transferrin receptor on normal venous endothelial cells [33]. 

Ultimately, a randomized phase III study of Tf-CRM107 was aborted because an intermediate analysis showed a response rate of 39%, an efficacy considered not significantly above standard-of-care treatments [90]. It has been hypothesized that Tf-CRM107′s failure is because the rapid cycling of transferrin in the cell limits its ability to deliver the diphtheria toxin. In vitro and in vivo studies have shown that mutant Tf, with a longer intracellular half-life, improves Tf-CRM107′s lethality [91]. It is also worth noting that CED studies of Tf-CRM107, consistent with many early CED trials, lacked measurements confirming the drug’s distribution through the tumoral and peritumoral space. 

#### 2.1.2. IL-4-Pseudomonas Exotoxin (NBI-3001/MDNA55)

Conceptually, NBI-3001 is similar to Tf-CRM107. The fusion protein consists of the catalytic domain of pseudomonas exotoxin A, which inhibits protein synthesis through the same mechanism as diphtheria toxin (i.e., the attachment of adenosine diphosphate ribose to EF2). Targeting is performed by the IL-4 moiety, which capitalizes on the observation that GBM tends to express IL-4R while normal brain does not [30].

In vitro studies showed that NBI-3001 was potently cytotoxic to human GBM cell lines at concentrations as low as 10 ng/mL [31]. NBI-3001 also induced dramatic tumor regression in a rodent flank model of GBM [32]. Based on these results, two phase I trials involving CED of NBI-3001 were conducted [29,34]. A pilot study performed by Rand et al. demonstrated the safety of NBI-3001 CED, with six of nine patients demonstrating extensive tumor necrosis, according to the study authors [29]. A second phase I study by Weber et al. showed similar results, along with a marginal improvement in overall survival in patients with recurrent glioblastoma (5.8 months with NBI-3001 vs. 4.7 months with surgical resection) [34]. 

Based on these early phase I successes, NBI-3001 was purchased by the pharmaceutical company Medicenna and christened MDNA55. A phase 2 study of MDNA55 was finished in 2019, and the full results were published in 2023 [84]. This well-designed study, conducted in patients with recurrent, IDH wildtype, nonresectable GBM, employed modern surgical planning alongside small-diameter catheters and used gadolinium as a marker of drug distribution. IL4 receptor expression was also quantified in patients with available biopsies. Median overall survival with a single treatment of MDNA55 was 10.2 months, which represented a significant increase over a literature-derived control value of 8.0 months. Perhaps unsurprisingly, overall survival was dependent on IL4 receptor status, with IL4R-high (by IHC) patients having a median survival of 15 months compared to 8.4 months in IL4R-low patients [84]. Medicenna is currently in the process of finding a commercial partnership for a planned phase III trial of MDNA55 (now rechristened bizaxofusp) [92]. 

#### 2.1.3. IL13-PE38QQR (Citredekin Besudotox)

Other than the terminated phase III study of Tf-CRM107, Citredekin Besudotox (CB) is the only agent to have been studied by a phase III clinical trial of CED. The conjugated toxin contains the same truncated pseudomonas exotoxin as NBI-3001/MDNA55 but attached to IL13, whose receptor, IL13R, is overexpressed in malignant gliomas compared to healthy brain [52,53]. GBM cell lines showed that CB had an IC_50_ ranging from <1–600 ng/mL, which correlated with IL13R expression and had limited cytotoxicity in normal human astrocyte lines [54].

Phase I trials of CB CED reported by Kunwar et al. and Vogelbaum et al. found 0.5 μg/mL of CB to be the maximum safely tolerated dose [51,55]. Kunwar et al. treated 51 patients and found a strong correlation between optimal catheter placement and overall survival, highlighting the importance of confirming successful drug delivery in any study of CED. Six patients also received ^123^I-HSA with PET imaging as a surrogate marker for CB distribution. Ten of 17 catheters assessed by ^123^I-HSA had a clinically significant volume of distribution, with deeply placed catheters (i.e., >20 mm from any surface) exhibiting the best performance [55]. Vogelbaum et al. demonstrated the safety of CB alongside temozolomide and radiation in newly diagnosed GBM. Notably, a marker of successful drug distribution was absent in this study [51]. 

The success of early clinical trials of CB led to CED’s only completed phase III clinical trial (PRECISE study), comparing post-resection CED of CB to Gliadel wafers in patients with recurrent GBM. Unfortunately, the PRECISE study failed to show a survival advantage of CB over Gliadel wafers [62]. Numerous explanations have been put forward for the study’s failure, chief among them that only 68% of catheters were placed according to the protocol guidelines, and no confirmation of successful drug distribution was performed. It is also worth noting that there was no quantification of IL13R expression in tumor tissue, despite some research suggesting that IL13R expression is not as uniform in gliomas as once thought [93]. The PRECISE trial makes clear the importance of using even imperfect surrogates for drug distribution. Additionally, studies involving targeted therapies would benefit from confirming the presence of their targets in patient glioma tissue.

#### 2.1.4. TP-38

TP-38 is a conjugated toxin that combines a modified pseudomonas exotoxin to transform growth factor-α, a ligand that binds epidermal growth factor receptor (EGFR). EGFR is overexpressed in 60–80% of glioblastomas and has a relatively low level of expression in normal brain tissue [60,61,94].

Despite a lack of published preclinical in vivo studies, TP-38 was used by Sampson et al. in a phase I clinical trial of TP-38 CED in 20 patients [59]. While intracerebral TP-38 was well-tolerated, the study once again highlighted the importance of catheter placement in obtaining a clinically relevant volume of drug distribution. Co-infusion of TP-38 (44 kDa) with ^123^I-HSA (66.5 kDa) in eight patients revealed a significant number of cases were complicated by leakage into the subarachnoid space (44%) or pooling in the resection cavity (25%), thereby preventing a large volume of distribution in the brain parenchyma. In contrast, successful infusion into the intraparenchymal space was only seen in 19% of TP-38 infusions [59]. The study of TP-38, similar to Citredekin Besudotox above, highlights that ensuring adequate intraparenchymal drug distribution is essential before drawing any conclusions about the efficacy of an agent given by CED. 

### 2.2. Chemotherapies

The presence of relatively few studies on conventional, small-molecule chemotherapies delivered via CED is surprising since CED theoretically allows for the efficient distribution of almost any small molecule into the brain. To date, CED has only been used to deliver paclitaxel, topotecan, and carboplatin in GBM clinical trials. 

#### 2.2.1. Paclitaxel

Paclitaxel, a natural compound of the western yew, acts as a microtubule stabilizer, preventing the microtubule disassembly required during cell division. Millions of patients with a variety of solid cancers have been treated with paclitaxel, making it one of the most widely used and successful chemotherapies [95]. Early preclinical studies showed that paclitaxel was active against glioma cell lines and could prolong survival in glioma tumor-bearing rats [43,44]. Since paclitaxel penetrates the BBB poorly, it is a prime candidate for intratumoral delivery by CED.

The first phase I/II clinical trial of paclitaxel CED was done in 15 patients by Lidar et al. [42]. The first three patients received 7.2 mg/day for 5 days, with the remaining patients receiving 3.6 mg/day after a high rate of chemical meningitis was observed. Chemical meningitis remained a problem even with the dose reduction, and several other complications were reported, including wound dehiscence (and subsequent infections), which was attributed to subcutaneous leakage of paclitaxel. Also, rapid tumor necrosis in two patients required surgical debulking. Despite a high complication rate, a 73% response rate was observed, suggesting that paclitaxel is highly active against recurrent GBM. 

A similar phase I trial was performed by Tanner et al. with eight patients to investigate paclitaxel efflux along the catheter [56]. Interestingly, Tanner et al. found that sealing the burr hole following catheter placement led to a large increase in distribution volume (as estimated by DWI imaging) and necessitated a dose reduction to 1.8 mg/day to avoid neurotoxicity. It has been suggested that the presence of cremophore, an emulsifier that improves paclitaxel’s aqueous solubility, could lead to paclitaxel’s backflow along the catheter. Ultimately, conclusions regarding paclitaxel’s efficacy when delivered by CED are difficult to extract from the many independent variables, known and unknown, in early CED trials. Future studies of paclitaxel CED have to find ways to reduce drug efflux along the catheter path, perhaps through cremophore-free preparations, novel catheter designs, and modern surgical approaches [96].

#### 2.2.2. Topotecan

Topotecan is a derivative of camptothecin, a natural product of the deciduous Camptotheca tree native to China. Topotecan, like all camptothecin derivatives, is a topoisomerase I (TOPI) inhibitor. Topotecan creates a stable complex with DNA and TOPI, preventing the religation of DNA after TOPI creates a single-strand break as part of its physiologic role in unwinding DNA during replication. Eventually, the replication fork collides with the stable topotecan-DNA-TOPI complex, resulting in the generation of a double-strand DNA break, the accumulation of which leads to cell death [97]. Expression of topoisomerase genes, including TOPI, increases during cell proliferation and is correlated with Ki67 levels [98].

Topotecan CED has been shown to inhibit tumor proliferation and prolong survival in preclinical murine glioma models, and prolonged delivery is safe in pigs [66,67,83]. To date, two clinical trials of topotecan CED have been performed in adult patients with recurrent GBM [65,82]. The first, a phase I study of 16 patients, determined a maximally tolerated topotecan concentration of 0.1 mg/mL in a single 40 mL infusion [65]. Based on a high tumor response rate to a single infusion (11 of 16 patients), a phase Ib study of 5 patients was performed and recently completed [82]. This study utilized an implantable, refillable pump to deliver four cycles of topotecan to patients with recurrent GBM before the pumps were removed and the remaining tumor resected. While this study only involved five patients, the use of pre- and post-treatment biopsies allowed treatment response to be assessed outside of overall survival data. Topotecan CED has a clear antiproliferative effect, as measured by decreased Ki67 and SOX2 indices and is non-toxic to neurons, as measured by NeuN immunostaining [82]. The use of co-infused Gadavist in this trial, along with post-treatment biopsies, allowed the volume of topotecan distribution to be approximated and tissue topotecan concentrations to be measured directly after treatment. 

Topotecan was the first drug to be administered by CED to pediatric patients with diffuse midline gliomas (DMG) [99]. Serial MRIs of the two pediatric patients who received topotecan CED showed a modest reduction in tumor size, but the high initial infusion rate was poorly tolerated. Nevertheless, CED into the brainstem was shown to be feasible, opening the door for future pediatric CED studies. To date, trials of radiolabeled antibodies, IL13-pseudomonas toxin, oncolytic viruses, and MTX110 (aqueous panobinostat) CED have been published for pediatric DMG [100,101,102,103].

Finally, a clinical trial (NCT02022644) involving nanoliposomal-irinotecan (Onivyde) was recently completed, and its results are pending publication. Similar to topotecan, irinotecan is a camptothecin derivative whose active metabolite (SN-38) inhibits topoisomerase I.

#### 2.2.3. Carboplatin

Following cisplatin’s approval by the FDA in 1978 for the treatment of testicular and ovarian cancer, carboplatin was developed as a less toxic cisplatin derivative [104]. The dicarboxylate-cyclobutane moiety on carboplatin is more stable than cisplatin’s two chloride groups, decreasing its nephrotoxicity while maintaining cisplatin’s antineoplastic effects in several mouse tumor models. The cytotoxic mechanism of platinum drugs is complex, but all platinum drugs bind DNA, creating intra- and inter-strand DNA crosslinks that lead to wide-ranging toxic consequences [105].

Based on in vitro sensitivity studies, systemically delivered carboplatin and bevacizumab were trialed in patients with recurrent GBM, but carboplatin was found to increase systemic toxicity with no improvement in tumor response rate or overall survival [73,74]. Drug concentration studies have shown that IV platinum drugs poorly penetrate brain tumor tissue, making them good candidates for CED [75]. Based on a survival benefit in a preclinical rodent glioma model, a phase I study of carboplatin CED was planned [76,106]. Unfortunately, very low doses of carboplatin were used in this trial (up to 74 ng/mL in humans vs. 2 mg/mL in the preclinical rat model), which limits any conclusions regarding the efficacy or safety of carboplatin CED [72]. Further studies are needed to determine the maximally tolerated dose of carboplatin CED and elucidate its potential efficacy in human GBM. 

#### 2.2.4. Mitoxantrone

Trials involving the CED of mitoxantrone, a topoisomerase II inhibitor, are limited. A preliminary report from Italy in 2005 reported safe CED of mitoxantrone in a preliminary study of 12 patients but gave limited data [46]. Subsequent reports from the same researchers focused on the locoregional delivery of mitoxantrone in the surgical resection cavity via a Rickam reservoir, an idea separate from CED [107].

### 2.3. Immunotherapy

Creating a cytotoxic or “hot” immune environment is the current goal of much oncological research. CAR T-cell therapy against GBM has engendered some recent excitement [108]. Still, efforts to reverse the “cold” tumor microenvironment of GBM have been largely unsuccessful, partly due to the very low number of tumor-infiltrating lymphocytes and low mutational burden in GBM. Past attempts to deliver immunotherapies by CED have been unsuccessful, but a current CED trial is ongoing that combines a conjugated toxin (D2C7) with an anti-CD40 monoclonal antibody (NCT05734560).

#### 2.3.1. Trabedersen

Trabedersen is an oligodeoxynucleotide that targets TGFB2 RNA, blocking the immunosuppressive effects of TGF-β [58]. TGF-β exerts its immunosuppressive effects through several mechanisms, including suppression of NK-cells and CD8+ T-cells and upregulation of Foxp3+ T-regulatory cells. TGF-β has also been implicated in helping drive glioma progression through uncertain pathways, although it certainly promotes a pro-tumoral immune microenvironment [109]. Preclinical studies of trabedersen demonstrated that it could reduce TGF-β secretion by approximately 50% and inhibit glioma cell migration in an in vitro spheroid model [57]. The most exciting in vitro result of trabedersen was a large increase in tumor cell lysis following coincubation with peripheral blood mononuclear cells [57]. It is worth noting, however, that additional IL-2 was required to activate the mononuclear cells, and the effect was highly variable, ranging from an increase of 41% to 521%. 

Despite the lack of published results examining trabedersen’s therapeutic effect in vivo, the CED of trabedersen was investigated in three different phase I/II clinical trials by Hau et al. [57]. Also, a large phase IIb trial was conducted by Bogdahn et al. of 145 patients with recurrent high-grade gliomas divided between standard chemotherapy, 10 μM, and 80 μM trabedersen [64]. In this trial, trabedersen CED did not lead to a significant benefit in their primary endpoint, tumor control rate at 6 months. The study authors did note, however, that 10 μM trabedersen did lead to a non-significant survival benefit compared to standard chemotherapy at 2 years (39% vs. 22%). Subgroup analysis found that this non-significant survival benefit was even more pronounced in the small sample (n = 12) of patients with recurrent grade III gliomas (83% vs. 42%). It was argued that the benefit of trabedersen derives from an immune response that builds over months and years and is, therefore, most relevant in less aggressive tumors [64]. Based on these results and other post hoc analyses, trabedersen was advanced to a phase III clinical trial that was ultimately terminated due to insufficient patient recruitment [110]. 

#### 2.3.2. Unmethylated CpG oligodeoxynucleotide (CpG-ODN)

Free, unmethylated CpG-ODNs are potent immunostimulators as they mimic bacterial DNA that lacks CpG methylation. Unmethylated CpG-ODNs activate Toll-like receptor 9 (TLR9), leading to a signaling cascade that ultimately activates NK, T, B, macrophage, and dendritic cells. Helper T cells are polarized towards TH1, which accelerates the development of an adaptive immune response [111]. Preclinical studies of unmethylated CpG-ODN showed that it could induce tumor clearance and long-term anticancer immunity in murine tumor models [48,49].

A phase I trial of CpG-ODN CED was done in 24 patients, which proved the feasibility of intratumoral delivery and established a phase II dose of 20 mg [47]. A subsequent phase II trial found that the treatment was generally poorly effective, although 15% of patients survived at least two years [63]. Both trials failed to approximate the volume of distribution and to quantify the TLR9 expression in patient tumors. Uneven volumes of drug distribution or unequal TLR9 expression could explain the variable responses seen in trial patients. Additionally, preclinical murine studies suggested that CpG-ODN depletes T-regulatory cells, the depletion of which may play a mechanistic role in tumor rejection [50]. Circulating T-regulatory cells were unchanged in patients treated with CpG-ODN CED, perhaps because the majority of patients were on steroids, which have been reported to increase circulating T-regulatory cells [112].

### 2.4. Viruses

The use of viruses to treat GBM is a potential alternative or adjunct to traditional immunotherapies. Oncolytic viruses not only directly lyse tumor cells but can also reverse GBM’s immunosuppressive microenvironment to promote greater T-cell infiltration. CED of oncolytic viruses has benefited small subsets of GBM patients, and current trials are trying to increase response rates by combining oncolytic viruses with immune checkpoint inhibitors.

#### 2.4.1. PVSRIPO

PVSRIPO is a live attenuated poliovirus vaccine (Sabin poliovirus vaccine) with the internal ribosome entry site (IRES) replaced with human rhinovirus’s IRES to avoid neurovirulence [113]. The efficacy of PVSRIPO is thought to be mediated through two mechanisms—the direct lysis of tumor cells and the activation of antigen-presenting cells (APCs). This is because PVSRIPO infection requires CD155, which is expressed on both tumor cells and (APCs). The benefit of tumor cell lysis is self-evident, while APC infection leads to the expression of type I interferon and inflammatory cytokines that promote T-cell stimulation in vitro [71].

A phase I trial of PVSRIPO via CED in 61 patients demonstrated the safety of PVSRIPO as well as a preliminary overall survival rate of 21% at 24 and 36 months [70]. Interestingly, a patient treated with lomustine 7 months after PVSRIPO exhibited rapid cystic degeneration of their tumor. It was hypothesized that a single cycle of lomustine led to a reduction in immunosuppressive regulatory T-cells, followed by reconstitution of effector T-cells. After this finding, 37 other patients were treated with chemotherapy post-PVSRIPO, with 11 exhibiting rapid cystic tumor degradation [70]. It is worth noting that some long-term survivors post-PVSRIPO did not receive lomustine. Nevertheless, these results were the basis for an ongoing phase II trial of PVSRIPO CED in combination with a single cycle of lomustine (NCT02986178). A phase II trial of PVSRIPO CED and systemic pembrolizumab is also ongoing (NCT04479241). 

#### 2.4.2. Reovirus

Reovirus is a widespread, asymptomatic virus naturally found in human gastrointestinal and respiratory tracts. It was discovered that fibroblasts, normally resistant to reovirus infection, became susceptible when transformed with activated Ras [114]. Reovirus resistance in most cells appears to be caused by the phosphorylation of ds-RNA-activated protein kinase (PKR) in the presence of viral transcripts. Phosphorylated PKR blocks viral protein translation by phosphorylating eukaryotic initiation factor 2, preventing the initiation of protein synthesis [115]. Ras activation blocks PKR phosphorylation, leading to reovirus susceptibility and cell lysis. Since activation of the Ras pathway has been implicated in a majority of high-grade gliomas, reovirus is an intriguing viral treatment that showed promise in a preclinical murine model [69].

Intratumoral reovirus injection was shown to be safe in recurrent GBM cases [116]. Based on this result, a phase I study of reovirus CED was performed with 15 patients [68]. While CED of reovirus was shown to be safe, the study lacked a way to measure virus distribution. Since positive patient responses were distributed over the range of viral doses, it is likely that many patients did not receive effective distribution volumes. This is of particular concern given the size of the reovirus (70–80 nm), although some studies have shown that 80 nm virus capsids are particularly mobile through the extracellular space [117,118]. Current investigations of the therapeutic potential of reovirus (e.g., in conjunction with anti-PD-1 antibodies) use IV delivery, as the virus appears to effectively cross the BBB and infect tumor cells when delivered intravenously [119]. 

#### 2.4.3. Delta24-RGD

Delta24-RGD (DNX-2401) is a replication-competent adenovirus designed for oncolysis. This is accomplished by making two fundamental modifications to the underlying adenovirus. First, base pairs are deleted in the E1A region, which prevents replication in cells with a functional Rb pathway but allows for replication in glioma cells with Rb pathway deficiencies. Also, the normal primary attachment site is substituted with αvβ3 and αvβ5 integrin receptors, whose corresponding ligands are expressed on glioma cells [78]. DNX-2401 was shown to potently lyse glioma cells in vitro and inhibit glioma growth in vivo in multiple rodent models [78,79,80,81]. 

A phase I study by Lang et al. in 25 patients demonstrated the safety of DNX-2401 when delivered intratumorally in recurrent GBM, with 5 patients surviving more than 3 years post-injection [120]. DNX-2401 CED was also studied, although a high rate of edema and viral meningitis was seen, which was thought to be because of suboptimal catheter placement and infusate backflow [77]. Similarly to studies with PVSRIPO, a subset of patients responded well to CED of DNX-2401, with tumor response and survival correlating with high post-treatment IFN-γ.

### 2.5. Miscellaneous

The ability of CED to administer a therapy directly to the tumoral space, with minimal distribution far from the tumor, lends itself to a variety of unique treatment paradigms. Radiolabeled antibodies, gene therapies, and even differentiation-based treatments have used CED to deliver their therapeutic agents.

#### 2.5.1. Liposomal HSV-tk

Few trials of CED better encapsulate the application of a novel treatment paradigm to glioblastoma than the use of the herpes simplex virus-thymidine kinase (HSV-tk) gene. The underlying strategy of HSV-tk treatment is elegant, even if the details of its implementation are daunting. The HSV-tk gene is inserted into GBM cells, which creates a novel susceptibility to subsequent ganciclovir treatment. The HSV-tk protein can phosphorylate ganciclovir, leading to a buildup of ganciclovir triphosphate in infected cells. The triphosphate form of ganciclovir is incorporated into growing DNA strands, where it prevents DNA elongation, leading to cell death [121]. 

Early attempts at HSV-tk transduction used retroviral vectors, which require mitotically active cells, creating a preference for GBM cell infection. Preclinical studies found that HSV-tk gene transduction was successful in creating a ganciclovir susceptibility both in vitro and in vivo [37,38,122]. The first clinical study of this paradigm placed xenografts of murine, HSV-tk vector-producing cells in the surgical resection cavity but achieved limited transfer of the HSV-tk gene [123].

Voges et al. were the first to use CED to deliver gene therapy to patients with recurrent GBM [35]. A cationic liposomal construct bearing the HSV-tk gene was used to improve HSV-tk transfer without the risk of immunogenicity associated with a viral construct [36]. Additionally, several studies demonstrated the efficacy of a cationic liposomal gene construct in murine glioma models [39,40,41]. A cationic liposome bearing the HSV-tk gene was infused alongside gadolinium as a surrogate for liposomal distribution. Four days after CED of the liposomal construct, IV ganciclovir was administered for two weeks. While Voges et al. demonstrated the safety of liposomal gene therapy, a therapeutic effect was only seen in a small area around the infusion site. This limited volume of distribution is likely because the size of the liposomal particles (180 ± 20 nm) prevents efficient movement through the extracellular space, whose pores are on the order of 60 nm [35,124].

#### 2.5.2. Cotara

Cotara is a ^131^I-labeled chimeric monoclonal antibody designed by Peregrine Pharmaceuticals that targets the intracellular H1-DNA complex present in all cells. The H1-DNA antigen is exposed in the necrotic core of GBM, where Cotara can bind and provide, in theory, a cytotoxic dose of radiation from its ^131^I moiety. In Patel et al., 51 patients received CED of Cotara with limited adverse events [45]. While Cotara is a novel therapeutic, the advantages of CED of radiolabeled antibodies over traditional brachytherapy modalities have yet to be demonstrated. While CED of Cotara is feasible, Peregrine stopped the development of Cotara after becoming Avid Biosciences and transitioning to manufacturing services [125].

#### 2.5.3. Human Recombinant Bone Morphogenic Protein 4 (hrBMP4)

Recent research has shown that bone morphogenic protein 4 (BMP4) can decrease glioma cell proliferation by targeting the CD133+ population of glioma cells that exhibit stem cell-like properties [86]. Conflicting results have been reported on the mechanism of BMP4, but it appears to be non-cytotoxic and likely involves some combination of promoting differentiation and blocking further proliferation [86,126]. Preclinical in vivo evidence for hrBMP4 as a GBM treatment is limited, but implantation of polyacrylic beads with adsorbed BMP4 led to a significant survival benefit in immunocompromised mice injected with dissociated human GBM cells [86].

A phase I trial evaluating CED of hrBMP4 in 15 patients with recurrent GBM was recently conducted in Europe by Bos et al. [85]. Two of the 15 patients had a durable response to hrBMP4 infusion. Because gadobutrol was co-infused with hrBMP4, the authors were able to determine that the majority of tumor recurrence after hrBMP4 treatment was outside areas of drug infusion. Tumor coverage, as measured by gadobutrol distribution, varied from 4 to 39%, but only 8.3% of tumor recurrence occurred inside the parenchymal volume treated with hrBMP4 [85]. Future studies of hrBMP4 will have to better determine the efficacy of this novel treatment paradigm and may benefit from post-treatment tissue analysis to better understand the molecular underpinnings of hrBMP4 treatment. Additionally, new strategies will be needed to better distribute hrBMP4 through the tumoral and peritumoral space. Nevertheless, targeting glioma stem cells with hrBMP4 represents exciting progress in treating the intratumoral heterogeneity of GBM.

## 3. Future Therapies and CED

While progress in CED’s technical and engineering challenges stands to advance its clinical impact, there is still a huge space of potential therapeutic compounds that have yet to be explored in human patients. Some of the more promising candidates are commented on below. 

### 3.1. Immunotherapies

Monoclonal antibodies (mAbs) are the most common immunotherapy currently used for peripheral organ tumors. Unfortunately, mAbs typically have poor BBB penetrance, and their use is often accompanied by off-target immune activation. CED of mAbs can, in theory, circumvent both problems. CED of agonistic CD40 mAbs, which induces activation of antigen-presenting cells and cytotoxic T-cells, has been effective in preclinical murine models [127]. Additionally, CED of CD40 mAbs may avoid some of the symptoms of cytokine release syndrome associated with systemic delivery [128].

CED of cytokines is another immunomodulatory strategy that may play a future role in GBM treatment. CED of IFN-γ has been shown to increase MHC expression, activate cytotoxic T-cells, and impair tumor growth in murine models [128].

### 3.2. Ferroptosis-Inducers

Ferroptosis is a non-apoptotic, iron-dependent cell death mechanism first described by Dixon et al. in 2012 [129]. Several studies have shown that cancer cells that are resistant to antimitotic agents tend to be susceptible to ferroptotic death [130,131]. This has led to the paradigm that rapidly proliferating cancer cells can be targeted by mitotic poisons, and quiescent cancer cells can be targeted by agents that induce ferroptosis. The presence of heterogeneous neoplastic cell subtypes is seen in glioblastoma, with quiescent populations of glioma cells overrepresented in recurrent GBM, suggesting a possible source of recurrence [132].

Recent research has shown quiescent, astrocyte-like glioma cell populations have a unique metabolic vulnerability to GPX4 inhibition, which induces ferroptosis [133]. While GPX4 inhibition via RSL3 led to a non-significant survival benefit in a murine MG3 tumor model, a significant survival benefit was seen when GPX4 inhibition was combined with cysteine and methionine restriction, suggesting a future role for ferroptotic agents may exist in the treatment of malignant gliomas [134].

### 3.3. Epigenetic Drugs

GBM was one of the first tumors where an epigenetic modification, MGMT methylation, was of clinical significance [135]. Additionally, in 2012, the majority of diffuse midline gliomas, GBM’s pediatric cousin, were discovered to have H3.3 mutations with subsequent epigenetic consequences (K27M in midline gliomas, G34R/V in pediatric hemispheric gliomas) [136]. While histone mutations are significantly rarer in adult gliomas, epigenetic changes have also been found to drive glioma cell stemness, with adaptive chromatin remodeling allowing glioma cells to exhibit a stem cell-like phenotype in response to selective pressures [137]. Finally, IDH mutation and the subsequent production of 2-hydroxyglutarate have been linked to epigenetic dysregulation and impaired cell differentiation [138]. While a more thorough review of the epigenetic targeting of glioblastoma can be found elsewhere, several intriguing epigenetic drugs may stand to benefit from CED.

**Figure 1:**
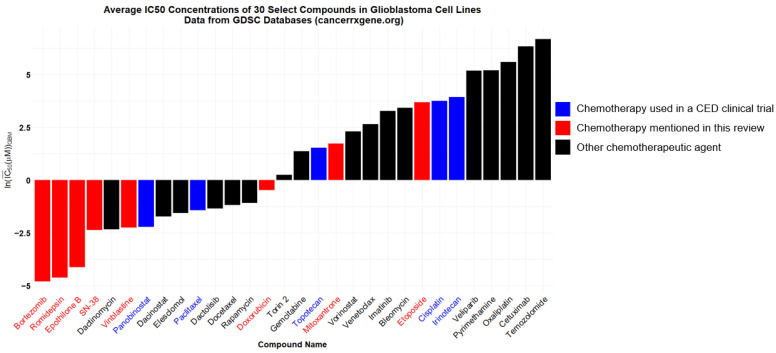
This figure depicts the average IC_50_’s in micromolar of select compounds against glioblastoma cell lines. Bars represent the natural logarithm of the arithmetic average of IC_50_’s in micromolar across 20+ GBM cell lines present in the Cancerrxgene database, an online database of cell line sensitivities to various drugs. Blue bars represent the compounds used in CED clinical trials. Red bars represent the compounds mentioned in this review that have not yet reached clinical trial via CED.

Histone demethylase inhibitors were initially developed in an attempt to address decreased methylation in pediatric K27M gliomas, and the broad demethylase inhibitor JIB 04 shows promising synergy with TMZ and counteracts TMZ resistance in GBM cells in vitro [139]. Also, several histone deacetylase (HDAC) inhibitors have shown potent in vitro effects against glioma cells (Figure 1) [140]. HDAC inhibitors may shift glioma cells away from the “stem cell” phenotype and provide synergistic benefits when combined with traditional chemotherapies [141].

Romidepsin and panobinostat are FDA-approved HDAC inhibitors and were trialed against glioblastoma via systemic delivery and found ineffective despite a marked lack of evidence that they can cross the BBB to a significant degree [142,143]. Interestingly, a phase II study demonstrated that the addition of valproic acid, which, among a multitude of functions, inhibits HDAC and crosses the BBB, to standard-of-care treatment led to some favorable patient outcomes [144]. Also, it is worth noting that aqueous panobinostat was successfully given by CED to seven newly diagnosed pediatric diffuse midline gliomas (DMG) in a recent phase I study, with a median overall survival of 26 months in the small study [102]. Another study of aqueous panobinostat CED for DMG was completed in 2023, and the results are pending publication (NCT04264143). Both romidepsin and panobinostat stand to benefit from CED trials in the short term, and further research refining their therapeutic mechanism and investigating their effects alongside traditional antimitotic agents is warranted.

### 3.4. Chemotherapies

Only three conventional chemotherapies (paclitaxel, topotecan, and carboplatin) have been explored in human CED clinical trials. Since CED allows for the delivery of both targeted therapies and broadly cytotoxic agents without concern for BBB penetrance or systemic toxicity, a wide range of cytotoxic compounds, both FDA-approved and experimental, can be trialed via CED with a reasonable expectation of success. Even a cursory examination of glioma cell lines’ sensitivities to different chemotherapeutics shows potential agents worthy of preclinical CED investigation (Figure 1).

As reviewed above, carboplatin CED has been the subject of one published clinical trial but is worthy of further investigations at physiologically relevant doses. Additionally, while topotecan CED has been investigated in clinical trials, the topoisomerase II (TOPII) inhibitor etoposide also shows promising antitumoral effects when given by CED in murine models of proneural gliomas [145]. It is worth noting that the anthracycline berubicin, a TOPII inhibitor that crosses the BBB, had promising phase I results and is currently under investigation in a phase II trial (NCT04762069).

The results of paclitaxel CED trials suggest that future investigation of microtubule drugs may be warranted. While paclitaxel’s solubility and technical factors contributed to a high rate of complications, the high patient response rate suggests that drugs with similar mechanisms of action, such as vinblastine or epothilone derivatives, could play a future role in treatment when delivered by CED. Epothilone derivatives are especially intriguing candidates for CED delivery as they are water soluble with likely poor BBB penetration.

In addition to antimitotic agents, targeted chemotherapeutics will likely have a role in future CED studies. Targeting the EGFR, mTOR, Rb, and p53 pathways are all ideas under current clinical investigation via systemic drug delivery [146]. Any targeted therapy with dubious BBB penetration is a potential candidate for study through CED. For instance, bortezomib, a proteasome inhibitor with in vitro efficacy against many glioma cell lines (Figure 1), likely poorly penetrates the BBB [147].

## 4. Conclusions

The history of CED clinical trials winds its way through conjugated-toxin therapies (e.g., MDNA55, CB), select chemotherapeutics (e.g., paclitaxel and topotecan), exotic immunomodulators (e.g., trabedersen and CpG UDN), and viruses (e.g., PVSRIPO and reovirus). While immunomodulators like trabedersen and CpG UDN appear to be of limited benefit, sweeping conclusions regarding the efficacy of most CED-trialed agents are difficult to make.

One theme that is readily apparent when reviewing CED of anticancer agents is that the CED platform itself must be weighed independently of the efficacy of the drug. Theoretically, CED is a powerful way to deliver almost any therapeutic directly to the tumoral and peritumoral parenchyma, but important technological challenges exist. 

Chief among these challenges is ensuring that CED provides an effective drug volume to each patient. Many early trials assumed this was the case, but later trials using gadolinium or radiotracers as markers of distribution have shown that significant variability in the volume of distribution exists between patients. Also, biopsies taken at catheter explant (as done by Spinazzi et al.) offer another novel method to measure drug concentrations distant from the catheter tip [82]. Reducing backflow along the catheter and managing heterogeneity in the porosity of the extracellular matrix are also important technological challenges. 

Conclusions regarding the efficacy of CED-trialed drugs are confounded by a number of factors. Trials to date have focused almost exclusively on recurrent high-grade gliomas in adults, and it is unknown if CED would prove more effective against primary GBM or low-grade gliomas. Some recent phase I trials have investigated CED in pediatric DMG patients, and further investigations with larger pediatric cohorts are warranted. Additionally, variable volumes of distribution, tumor heterogeneity, and poor catheter placement have confounded CED trials. As noted above, even the failure of the phase III PRECISE trial (Cetredekin Besudotox) is difficult to attribute to the agent itself, given the myriad of problems that occurred with catheter placement. To draw meaningful conclusions about the efficacy of therapeutics given by CED, it is essential for future studies to include markers of drug distribution and novel ways of determining tumor response to a drug. This will likely require focused trials with a limited number of highly analyzed patients. Novel methods of assessing tumor response are needed as almost every CED trial to date has lacked the power to draw meaningful conclusions about overall survival. Also, trials with targeted therapies would benefit from quantifying the presence of the drug target in patient tumors. 

Refillable, long-term CED pumps represent an important advancement in CED technology. They theoretically allow for treatments to be used in sequence, akin to how many peripheral cancers have first-, second-, and third-line therapies. In the future, CED of sequential and combination therapies will likely be necessary to target the well-described heterogeneity of glioblastomas. Non-CNS tumors are increasingly treated with chemotherapeutic combinations, and similar approaches are likely needed in the CNS.

CED is an invasive and expensive approach to drug delivery in the CNS, but the devastating nature of high-grade gliomas warrants aggressive intervention. Few other approaches can deliver such a wide range of therapeutic agents directly to tumor tissue while avoiding systemic toxicity. Nevertheless, the history of CED highlights that technological issues must be evaluated separately from the efficacy of therapeutic agents. As CED technology and clinical trial design improve, future conclusions regarding drug efficacy will be easier to draw. 

## Figures and Tables

**Table 1:** Chronological summary of adult GBM CED clinical trials along with preclinical evidence supporting their therapeutic agent.

First Author (Year)	Phase	Tumor Type	Therapeutic Agent	Agent Type	Vi	Infusion Rate	Dose	# of Catheters	Markers of Vd	n	Preclinical Studies
Laske (1997) [25]	I	rGBM, rAA, Mets	Transferrin-CRM107 (Tf-CRM107)	Conjugated toxin	5–180 mL	0.24–0.6 mL/hr	0.5–199 μg	1–3	None	15	[26,27,28]
Rand (2000) [29]	I	rGBM	IL4-pseudomonas exotoxin (NBI-3001/MDNA55/bizaxofusp)	Conjugated toxin	30–185 mL	0.3–0.6 mL/hr	6–720 μg	1–3	None	9	[30,31,32]
Weaver (2003) [33]	II	rGBM, rAA	Tf-CRM107	Conjugated toxin	40 mL × 2	0.2 mL/hr	27 μg	1–3	None	44	[26,27,28]
Weber (2003) [34]	I	rGBM, rAA	IL4-pseudomonas exotoxin (NBI-3001/MDNA55/bizaxofusp)	Conjugated toxin	40–100 mL	0.4–1 mL/hr	240–900 μg	1–3	None	27	[30,31,32]
Voges (2003) [35]	I	rGBM	HSV-tk	Liposomal gene therapy	3.5 mL	0.6 mL/hr	See [36]	1–2	Coinfused Magnevist in 6 of 8 patients	8	[37,38,39,40,41]
Lidar (2004) [42]	I/II	rGBM, rAA	Paclitaxel	Chemotherapy	30 mL	0.3 mL/hr	18–36 mg	1	DWI imaging	15	[43,44]
Patel (2005) [45]	I/II	pGBM, rGBM, rAA	Cotara ^131^I-mab	Radio-labeled antibody	4.5–18 mL	0.18 mL/hr	1–1.5 mCi/cc	1–2	^131^I	51	-
Boiardi (2005) [46]	I	rGBM	Mitoxantrone	Chemotherapy	-	-	-	-	None	12	-
Carpentier (2006) [47]	I	rGBM	CpG oligo-nucleotide	Immunotherapy	1 mL	0.2 mL/hr	0.5–20 mg	1–2		24	[48,49,50]
Vogel-baum (2007) [51]	I	pGBM, AOA	cintredekin besudotox (interleukin-13-PE38QQR)	Conjugated toxin	72 mL	0.75 mL/hr	18–36 μg	2–4	None	22	[52,53,54]
Kunwar (2007) [55]	I	rGBM	cintredekin besudotox (interleukin-13-PE38QQR)	Conjugated toxin	72 mL	0.75 mL/hr	18–72 μg	1–3	^123^I-HSA in 6 of 51 patients	51	[52,53,54]
Tanner (2007) [56]	I	rGBM	Paclitaxel	Chemotherapy	36 mL	0.3 mL/hr	9–18 mg	1–2	DWI imaging	8	[43,44]
Hau (2007) [57]	I/II	rGBM, rAA	AP12009/Trabedersen (TGF-β2 inhibitor)	Immunotherapy	23–80.5 mL	0.24–0.48 mL/hr	0.354–39.62 mg	1	None	24	[57,58]
Sampson (2008) [59]	I	rGBM, GSC, rAO	TP-38	Conjugated toxin	40 mL	0.8 mL/hr	1–4 μg	2	^123^I-HSA in 8 of 16 patients	20	[60,61]
Kunwar (2010) [62]	III	rGBM	cintredekin besudotox (interleukin-13-PE38QQR)	Conjugated toxin	72 mL	0.75 mL/hr	36 μg	1	None	296	[52,53,54]
Carpentier (2010) [63]	II	rGBM	CpG oligo-nucleotide	Immunotherapy	2 mL	0.2 mL/hr	20 mg	2	None	34	[48,49,50]
Bogdahn (2011) [64]	IIb	rGBM, rAA	AP12009/Trabedersen (TGF-β2 inhibitor)	Immunotherapy	40.3 mL × 1–11	0.24 mL/hr	2.48–19.81 mg	2	None	145	[57,58]
Bruce (2011) [65]	Ib	rGBM, rAA, rAO, rAE	Topotecan	Chemotherapy	40 mL	0.2 mL/hr	0.8–5.32 mg	2	None	15	[66,67]
Kicielinski (2014) [68]	I	rGBM, rAA	Reovirus	Virus	30 mL	0.4 mL/hr	10^8^–10^9^ TCID	2–4	None	15	[69]
Desjardins (2018) [70]	I	rGBM	PVSRIPO	Virus	3.25 mL	0.5 mL/hr	10^7^–10^9^ TCID	1	None	61	[71]
Wang (2020) [72]	I	rGBM, rOG	Carboplatin	Chemotherapy	54 mL	0.75 mL/hr	1–4 μg	2–3	None	10	[73,74,75,76]
van Putten (2022) [77]	I	rGBM	Delta24-RGD (DNX-2401)	Virus	20 mL	0.2–0.3 mL/hr	10^7^–10^11^ vp	4	None	19	[78,79,80,81]
Spinazzi (2022) [82]	Ib	rGBM	Topotecan	Chemotherapy	9.6 mL × 4	0.2 mL/hr	0.64 mg × 4	1	Coinfused Gadavist	5	[66,67,83]
Sampson (2023) [84]	IIb	rGBM	IL4-pseudomonas exotoxin (NBI-3001/MDNA55/bizaxofusp)	Conjugated toxin	12–66 mL	0.58–2.83 mL/hr	18–240 μg	1–4	Coinfused Magnevist	47	[30,31,32]
Bos (2023) [85]	I	rGBM	rhBMP4	Differentiation protein therapy	44–66 mL	0.153–0.456 mL/hr	0.5–18 mg	3	Coinfused Gadobutrol	15	[86]

Abbreviations: Vi—Volume infused; rGBM—recurrent glioblastoma (WHO Grade IV); rAA—recurrent anaplastic astrocytoma (WHO Grade III); rAO—recurrent anaplastic oligodendroglioma; rAE—recurrent anaplastic ependymoma; rOG—recurrent oligodendroglioma; AOA –anaplastic oligoastrocytoma; GSC—gliosarcoma; pGBM—primary glioblastoma; TCID—tissue culture infectious doses; vp—viral particles; HSA—human serum albumin; mCi—millicurie; cc—cubic centimeter; MTC—mean tumor coverage; Vd—volume of distribution; #—number; n—number of patients.
